# Eclectic Department

**Published:** 1854-01

**Authors:** 


In the Peter Paul Hospital of St. Petersburg, in Russia, occurred a case of atrophy of the heart, where the organ was only two and one-half inches long, and two inches wide, and the pericardium contained one pound of clear serum. The patient, 35 years old, had died of general dropsy, the urine containing a considerable amount of albumen, while the kidneys were found normal.— *Mediz. Zeitung, Russland's*, 1852, *No.* 27.

Dr. Heinrich denies, according to his experience, benefit being derived from paracentesis, where there is considerable amount of exudation in the pleura, or in the pericardium, but convinced himself, in such conditions, of the great efficacy of an infus. flor. arnicæ with tartar emetic.— *Mediz. Zeitung, Russland's*, 1852, *No.* 30.

